# What does it take to be healthy? Investigating immune-deficiency in non-immunodeficient scenarios

**DOI:** 10.1186/1479-5876-10-S3-I8

**Published:** 2012-11-28

**Authors:** Elisa Binda, Reiner Schulz, Ania Showera, Andrew Cope, Michael Malim, John Cason, Mark Peakman, Adrian Hayday

**Affiliations:** 1 King’s College London School of Medicine, Guy’s Hospital, London, UK

## 

Viral pandemics can arise rapidly and unexpectedly, necessitating the prompt development and administration of relevant vaccines. Nonetheless, despite our impressive knowledge of immunological pathways, cell-types, molecules, and pathways, we have little understanding of how effective, integrated immune responses to vaccination are composed. Indeed, we have conspicuously poor understanding of normality in human immune function, and hence few effective measures of it [1]. Our study was designed to redress this situation. Longitudinal molecular, cellular, and functional immune-monitoring was undertaken in a cohort of >150 healthy volunteers, resident in the UK, either side of a richly adjuvanted H1N1 vaccine, with blood sampling at day -7; day 0 [vaccine]; day +1; day +7; day +14; day +63. The data to be presented will reflect gene expression profiling in peripheral blood mononuclear cells, multiplex detection of serum cytokines and chemokines and cell phenotyping, as reconciled with initial clinical phenotype [fever/adverse events [AE] *vs* asymptomatic] and downstream outcome measures. The results attest to the practicality of immune-monitoring, and its capacity to provide new knowledge concerning the stability and dynamics of human immune measures. Of note, gene expression profiles diverged overtly at day +1, according to symptoms, but largely re-converged by day +7 to a B cell-rich response. Specific genes were associated with AE, but these were not those that were anticipated a *priori*. Highly significant changes in numerous serum inflammatory markers were detected at day +1. Hence, the study highlights the potential of novel markers of immune responsiveness. The study likewise permits the search for specific markers and overall immune profiles that predict the functional response to vaccination. The intrinsic content of such studies, and their comparison with similar studies conducted elsewhere, help establish the normal range of human immune function and should thereby facilitate the identification of immune pathology. At the same time, logistical and other challenges relating to immune monitoring will be discussed.
